# New species of *Limnophyes* Eaton (Diptera, Chironomidae) from China and synonymy of *L. fuscipygmus* Tokunaga, 1940

**DOI:** 10.3897/zookeys.1011.58993

**Published:** 2021-01-18

**Authors:** Wenbin Liu, Cong Zhao, Fanqing Kong, Chuncai Yan, Xinhua Wang

**Affiliations:** 1 Tianjin Key Laboratory of Conservation and Utilization of Animal Diversity, Tianjin Normal University, Tianjin, 300387, China Tianjin Normal University Tianjin China; 2 Ecological Environment Monitoring and Scientific Research Center of Haihe River Basin and Beihai Sea Area, Ministry of Ecological Environment, Tianjin, 300170, China Ecological Environment Monitoring and Scientific Research Center of Haihe River Basin and Beihai Sea Area, Ministry of Ecological Environment Tianjin China; 3 College of Life Science, Nankai University, Tianjin 300071, China Nankai University Tianjin China

**Keywords:** Identification key, morphology, synonymy, systematics, taxonomy

## Abstract

Two new species, *L. minerus* Liu & Yan, **sp. nov.** and *L. subtilus* Liu & Yan, **sp. nov.** are described and illustrated as adult males. *Limnophyes minimus* (Meigen, 1818) is assigned as a senior synonym of *L. minerus* Tokunaga, 1940. A key to males of *Limnophyes* from China is presented.

## Introduction

*Limnophyes* was erected by Eaton (1875) with *Limnophyes pusillus* Eaton, 1875 as type species. Sæther (1990a, b) reviewed the Holarctic, Afrotropical and Neotropical members of this genus. Subsequently, the genus in different life stages and geographical areas were studied by a number of authors (Wang 2000; Chaudhuri et al. 2001; Langton and Moubayed 2001; Yamamoto 2004; Makarchenko et al. 2005; Mendes et al. 2007; Murray 2007; Makarchenko and Makarchenko 2011; Moubayed 2011; Pinho and Andersen 2015; Song et al. 2020). To date, the genus comprises more than 90 species recorded worldwide (Ashe and O’Connor 2012; Song et al. 2020). However, although 28 species of *Limnophyes* have been recorded in Japan, only four of these are widespread Holarctic species. Most likely, many of the remaining Japanese names are synonymous with names from other regions, and a revision is desirable (Przhiboro and Sæther 2007).

To date, 16 species of the genus from China were described or recorded including two larvae, *L. pentaplastus* (Kieffer, 1921) and *L. pumilio* (Holmgren, 1869) (Wang and Sæther 1993; Wang 1997; Wang 2000; Song et al. 2020).

In this study we describe two new species of *Limnophyes* from Oriental China as male adults, provide a key to the known adult males of the genus from China, and suggest that *L. fuscipygmus* Tokunaga, 1940 from China be considered a synonym of *L. minimus* (Meigen, 1818).

## Methods and material

The morphological nomenclature follows Sæther (1980). The material examined was mounted on slides following the procedure outlined by Sæther (1969). All samples have been stored in 85% ethanol prior to preparation. Color is described as observed in specimens mounted in Canada balsam on slides. Measurements are given as ranges followed by the arithmetic mean when four or more species were measured, followed by the number of specimens measured (*N*) in parentheses. All types are deposited in the College of Life Sciences, Nankai University, China (**BDN**).

Abbreviations used in text as follows: AR, antennal ratio = length of ultimate flagellomere/combined lengths of flagellomeres one to penultimate; fe, femur; HR, hypopygium ratio = gonocoxite length/gonostylus length; HV, hypopygium value = body length/gonostylus length × 10; LR, leg ratio = tarsomere length/tibia length; LR_1_, tarsomere I length/tibia length; p1–3, Legs (1–fore, 2–mid, 3–hind); R, Radius; R_1_, Radius 1; R_4+5_, Radius four and five; Cu_2_, the second Cubitus; Ta1–5, tarsomeres 1–5; Ti, tibia; VR, ratio of length of Cu/length of M; BV, Length of (femur + tibia + ta1) / length of (ta2 + ta3 + ta4 + ta5); SV, Length of (femur + tibia) / length of ta; BR, longest seta on tarsomere 1/minimum width of tarsomere 1.

## Taxonomy

### Limnophyes minerus

Taxon classificationAnimaliaDipteraChironomidae

Liu & Yan sp. nov.

6A2E82F7-8478-5B17-BD7C-4DF84A149D0C

http://zoobank.org/559838A7-DBA7-4578-8408-841097D8A54F

Figs 1–4

#### Type material.

***Holotype*** male (BDN No. 13105), China: Sichuan Province, Kangding County, Wasi River, 30.051°N, 101.964°E, 3124 m a.s.l., light trap, 15.vi.1996, X. Wang. ***Paratype***: 1 male, Sichuan Province, Daocheng County, Sangdui Town, 29.038°N, 100.297°E, 3447 m a.s.l., sweep net, 11.vi.1996, X. Wang; 1 male, Hubei Province, Wufeng County, Houhe River, 30.199°N, 110.675°E, 1723 m a.s.l., sweep net, 1.vii,1997, B. Ji.

#### Diagnostic characters.

The species can be separated from other members of the genus by having minute inferior volsella, virga consisting of one tapering spine, no lanceolate setae, and AR 0.24–0.27.

#### Etymology.

From the Latin, *minerus*, minute or tiny, referring to the reduced inferior volsella, adjective in the nominative singular.

#### Description.

Male (*N* = 3).

Total length 1.68–1.80 mm. Wing length 0.95–1.25 mm. Total length / wing length 1.44–1.76. Wing length / length of profemur 2.57–3.20.

***Coloration*.** Head and thorax dark brown. Abdomen and legs brown. Wing nearly transparent.

***Head*.** Antenna with 13 flagellomeres. AR 0.24–0.27. Ultimate flagellomere 79–98 µm long. Temporal setae 4–5, including 1 inner vertical, 1–3 outer verticals and 1–2 postorbitals. Clypeus with 10–21 setae. Tentorium 110–120 µm long, 14–18 µm wide. Palpomere lengths (in µm): 17–20, 22–25, 42–55, 35–52, 79–95. Length ratio of palpomeres 5/3 1.73–1.91.

**Figures 1–4.**
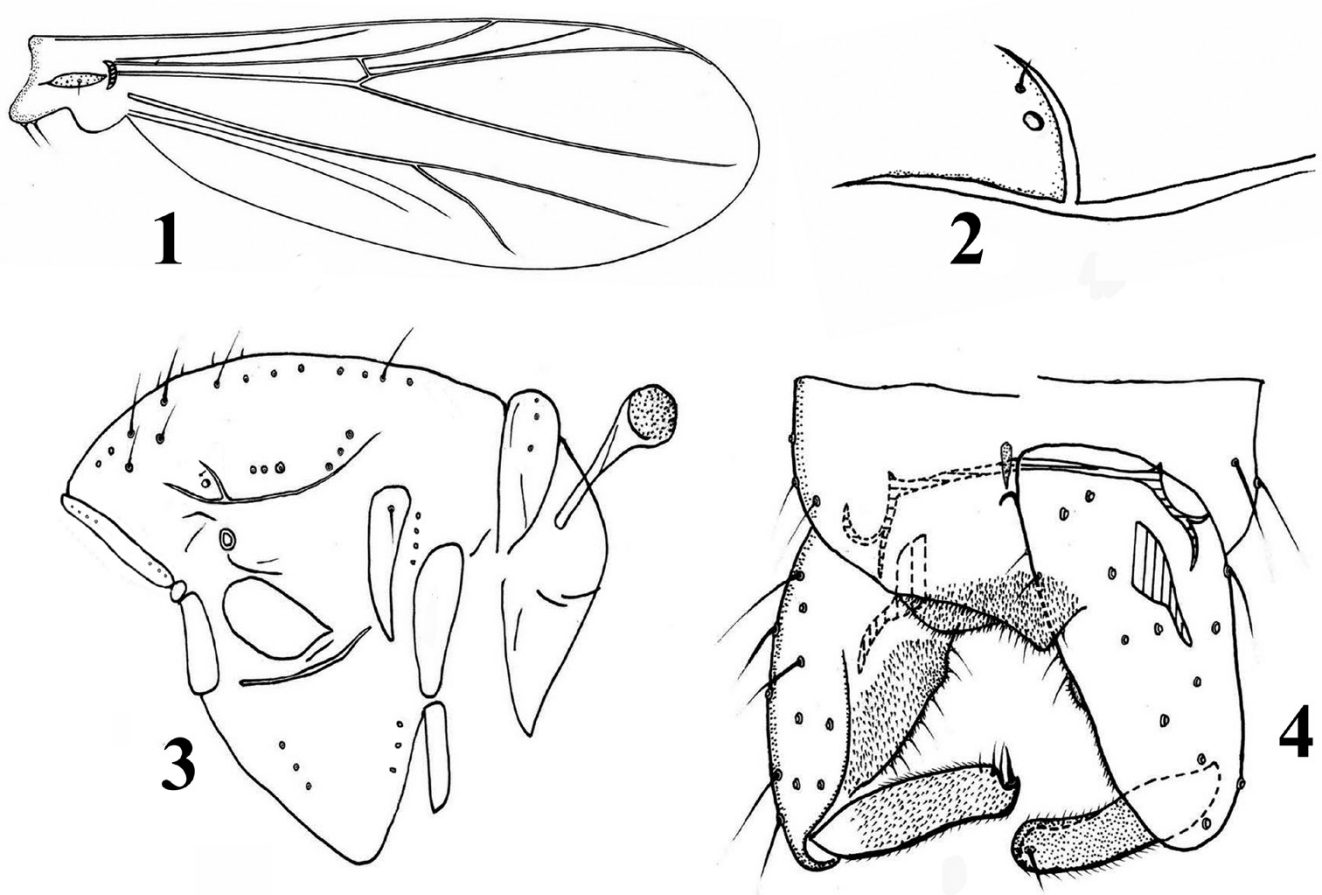
*Limnophyes minerus* Liu & Yan, sp. nov., male **1** wing **2** humeral area of the thorax **3** thorax **4** hypopygium.

***Wing*** (Fig. 1). Anal lobe reduced. VR 1.22–1.30. Brachiolum with one seta. R with 1–3 setae. Costal extension 40–50 µm long. Squama with 2–4 setae.

***Thorax*** (Figs 2, 3). Antepronotum with 2–4 median setae, and 2–3 lateral setae. Humeral pit (Fig. 3) small, with sclerotized anterior margin. Dorsocentrals 14–16, with 0–1 non-lanceolate humeral, and 14–15 non-lanceolate dorsocentrals. Preepisternum with 3 setae in anterior and posterior respectively. Acrostichals 4, prealars 4–5, supraalar 1, posterior anepisternum II with 3–4 setae, epimeron II with 4 setae, median anepistenum II with one seta; scutellum with 9 setae.

***Legs*** (*N* = 2). Spur of fore tibia 33–37 µm long, of mid tibia 17–23 µm and 13–18 µm long, of hind tibia 37–38 µm and 13–15 µm long. Setae of tibial comb 28–30 µm long, comb with 11 teeth. Width at apex of fore tibia 25–26 µm, of mid tibia 24–25 µm, of hind tibia 30 µm. Lengths and proportions of legs in Table 1.

***Hypopygium*** (Fig. 4). “Anal point” bluntly triangular, with 8–13 weak setae. Laterosternite IX with 2–3 setae. Phallapodeme 25–32 µm long; transverse sternapodeme 63–75 µm long. Virga 17–19 µm long, consisting of one tapering spine. Gonocoxite 113–125 µm long. Inferior volsella minute. Gonostylus 65–73 µm long, with pointed crista dorsalis. Megaseta 10–16 µm long. HR 1.55–1.89, HV 2.58–2.73.

**Table 1.** Lengths (in µm) and proportions of leg segments of male *L. minerus* Liu & Yan, sp. nov. (*N* = 3).

	fe	ti		ta_1_		ta_2_		ta_3_		ta_4_
p_1_	310–390	430–510		220–240		130–140		80–95		45–50
p2	380–420	370–470		150–200		85–100		40–60		40–45
p3	390–450	420–510		230–280		110–120		95–130		50
	ta_5_		LR		BV		SV		BR	
p1	45–60		0.47–0.52		3.20–3.34		3.36–3.75		2.20–2.25	
p2	35–50		0.43–0.47		3.92–5.45		4.45–5.00		2.00–2.33	
p3	45–60		0.55–0.56		3.41–3.47		3.43–3.52		2.50–2.67	

#### Remarks.

The new species can be separated from other members of the genus by having a minute inferior volsella. The characters of the new species closely resemble *L. ninae* Sæther, 1975 (Sæther 1975, 1990a). However, the new species differs from the latter on the basis of following characters: (1) the humeral pit of the new species lacking lanceolate prescutellars, whereas in *L. ninae* the humeral pit has lanceolate setae inside the margin; (2) virga consisting of three spines with the median much shorter in *L. ninae*, whereas the new species has a single virga; (3) the AR of new species (0.24–0.27) is much lower than in *L. ninae* (0.62–0.77).

#### Distribution.

The new species was collected in a subtropical mountain area in Hubei and Sichuan Provinces in Oriental China.

### Limnophyes minimus

Taxon classificationAnimaliaDipteraChironomidae

(Meigen, 1818)

18004C86-3BF2-559A-9E36-C6CEC21065F1

Chironomus minimus Meigen, 1818: 47.
Limnophyes pusillus Eaton, 1875: 60; Goetghebuer 1932: 196; 1940–50: 140; Brundin 1947: 83.
Spaniotoma (Limnophyes) pusilla (Eaton); Edwards, 1929: 355.
Limnophyes minimus (Meigen); Goetghebuer 1932: 108, 1940–50: 137.
Limnophyes interruptus Goetghebuer, 1938: 463.
Limnophyes immucronatus Sæther, 1969: 103.
Limnophyes hudsoni Sæther, 1975: 1032.
Limnophyes natalensis (Kieffer); Freeman 1957: 344, pro parte, (misidentified)
Limnophyes minimus (Meigen); Sæther 1990: 59; Wang and Sæther 1993: 216; Wang 2000: 636; Yamamoto 2004: 46; Makarchenko et al. 2005: 403, 2011: 115, Langton and Pinder 2007: 114.
Limnophyes fuscipygmus Wang, 2000: 636, *nec* Tokunaga, 1940: 287 syn. nov.

#### Material examined.

Fujian Province, Fuzhou Agricultural University Campus, sweep net, 5 males, 22.iv.1993, W. Bu; Fujian Province, Nanping County, Maodi Town, sweep net, 4 males, 22.ix.2002, Z. Liu; Fujian Province, Shanghang County, Mt. Buyun, light trap, 5 males, 6.v.1993, W. Bu; Fujian Province, Wuyishan Natural Reserve Area, light trap, 31 males, 25.iv.1993, W. Bu; Guangxi Autonomus Region, Jinxiu County, Luoxiang Town, sweep net, 2 males, 9.vi.1990, X. Wang; Guangxi Autonomus Region, Longsheng County, sweep net, 5 males, 24.v.1990, X. Wang; Guizhou Province, Daozhen County, Dashahe Natural Reserve Area, sweep net, 1 male, 22.v.2004, H. Tang; Guizhou Province, Daozhen County, Xiaoshahe River, sweep net, 1 male, 25.v.2004, H. Tang; Hebei Province, Chengde City, Beidai River, sweep net, 1 male, viii.1986, X. Wang; Hebei Province, Chengde City, Saihanba Forest Park, sweep net, 1 male, 15.vii.2001, Y. Guo; Henan Province, Luanchuan County, Longyuwan Park, sweep net, 1 male, 13.vii.1996, J. Li; Henan Province, Baiyunshan Forest Farm, sweep net, 3 males, 16.vii.1996, J. Li; Henan Province, Song County, Baiyunshan Forest Farm, sweep net, 3 males, 16.vii.1996, J. Li; Hubei Province, Hefeng County, Mt. Fenshui, light trap, 3 males, 16.vii.1997, B. Ji; Hubei Province, Lifeng County, Houhe River, sweep net, 2 males, 30.vi.1997, B. Ji; Hubei Province, Lichuan County, Mt. Xingdou, sweep net, 3 males, 30.vi.1997, B. Ji; Hubei Province, Xianfeng County, Pingbaying Park, sweep net, 3 males, 25.vi.1997, B. Ji; Jiangxi Province, Poyang Lake, sweep net, 4 males, 12.vi.2004, C. Yan; Jiangxi Province, Yifeng County, Mt. Gongshan, sweep net, 2 males, 8.vi.2004, C. Yan; Jiangxi Province, Wuyishan Natural Reserve Area, light trap, 2 males, 13.vi.2004, C. Yan; Ningxia Autonomous Region, Mt. Liupan, Erlonghe Forest Farm, sweep net, 6 males, 7.viii.1987, X. Wang; Shannxi Province, Ningshan County, Huoditang Forest Farm, sweep net, 2 males, 12.viii.1994, W. Bu; Sichuan Province, Ganzi City, Yajiang River, light trap, 2 males, 14.vi.1996, X.Wang; Sichuan Province, Kangding City, Wasi River, light trap, 2 males, 15.vi.1996, X.Wang; Sichuan Province, Litang County, Zhaisang Region, sweep net, 2 males, 11.vi.1996, X.Wang; Sichuan Province, Mt. Emei, sweep net, 1 male, 17.v.1987, X. Wang; Xizang Autonomus Region, Xiazayu County, sweep net, 2 males, 24.iv.1988, C. Deng; Xizang Autonomus Region, Shigatse City, Zhangmu Town, sweep net, 6 males, 18.ix.1987, C. Deng; Yunnan Province, Fumin County, Daying Town, sweep net, 1 male, 1.vi.1996, X. Wang; Yunnan Province, Wuding County, Mashan Town, sweep net, 1 male, 1.vi,1996, X. Wang; Zhejiang Province, Baishanzu Natural Reserve Area, light trap, 2 males, 18.iv.1994, H. Zou; Zhejiang Province, Tianmushan Natural Reserve Area, light trap, 1 male, 17.viii.1999, H. Zou.

#### Remarks.

*Limnophyes minimus* (Meigen, 1818) is one of the dominant species of *Limnophyes* in China. Of all the specimens examined, some variation can be found. One specimen from Guangxi Province had a strongly reduced anal lobe, antenna with 10 segments, and LR_1_ of 0.59, i.e., outside the range of 0.45–0.55. Two specimens from Sichuan and Yunnan provinces have AR 0.20 and 0.30, both lower than the minimum value of Sæther’s description (AR 0.48). One specimen from Fujian Province had 9-segmented antenna, and high length ratio of palpomeres 5/3 (2.11).

Tokunaga (1940) described the species *L. fuscipygmus* based on materials from Taiwan Province, China. The holotype specimen of *L. fuscipygmus* mainly agrees with the description of *L. minimus* by Sæther (1975: 1032, figs 2–4), especially the characters of humeral pit, anal point, inferior volsella, and gonostylus. Consequently, it must be considered a junior synonym of *L. minimus*.

#### Distribution.

The species is widespread, and it has been recorded in all the six Chinese geographical regions. It occurs both in Palaearctic and Oriental China.

### Limnophyes subtilus

Taxon classificationAnimaliaDipteraChironomidae

Liu & Yan sp. nov.

FEDC2DBD-80BB-594D-B442-7DBED161CD88

http://zoobank.org/56360531-A0B1-4140-8CB9-711A0E6672C7

Figs 5–8

#### Type material.

***Holotype*** male (BDN No. 12222), China: Sichuan Province, Daocheng County, Daocheng River, 29.112°N, 100.146°E, 2492 m a.s.l., sweep net, 11.vi.1996, X. Wang. ***Paratype***: 5 males, as holotype.

#### Diagnostic characters.

The new species can be separated from other members of the genus by having 9–21 lanceolate humerals, 7–9 lanceolate prescutellars, megaseta hair-like, virga very slender, and anal lobe moderately developed.

#### Etymology.

From the Latin, *subtilus*, thin, slender, referring to the shape of the virga, adjective in the nominative singular.

#### Description.

Male (*N* = 6).

Total length 2.53–2.83, 2.67 mm. Wing length 1.56–1.73, 1.63 mm. Total length / wing length 1.54–1.74, 1.64. Wing length / length of profemur 2.97–3.16, 3.05.

***Coloration*.** Head, thorax and legs dark brown. Abdomen yellowish brown. Wing nearly transparent.

***Head*.** Antenna with 13 flagellomeres. AR 0.76–0.88, 0.83. Ultimate flagellomere 295–330, 310 µm long. Temporal setae 5–7, 6, including 1 inner vertical, 2 outer verticals and 2–4, 3 postorbitals. Clypeus with 14–18, 16 setae. Tentorium 132–140, 136 µm long, 22 µm wide. Palpomere lengths (in µm): 25–31, 28; 40–45, 43; 79–95, 86; 78–90, 85; 120–128, 125. Length ratio of palpomeres 5/3 1.26–1.62, 1.44.

**Figures 5–8.**
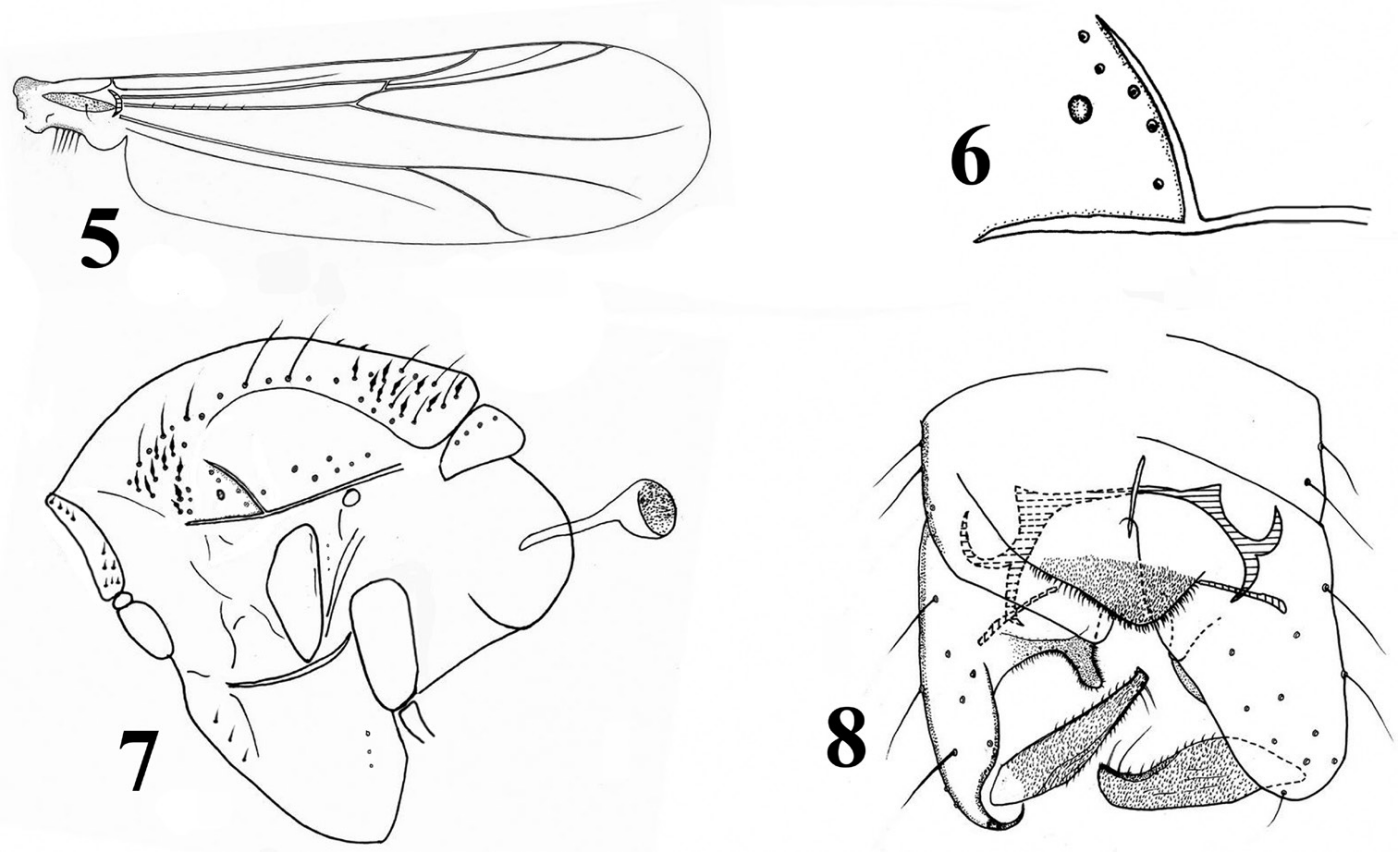
*Limnophyes subtilus* Liu & Yan, sp. nov., male **5** wing **6** humeral area of the thorax **7** thorax **8** hypopygium.

***Wing*** (Fig. 5). Anal lobe moderately developed. VR 1.20–1.29, 1.26. Brachiolum with one seta. R with 5–6, 5 setae. Costal extension 22–40, 31 µm long. Squama with 6–8, 7 setae.

***Thorax*** (Figs 6, 7). Antepronotum with 3–4, 4 median and 4–6, 5 lateral setae. Humeral pit (Fig. 7) rounded, with sclerotized posterior margin. Dorsocentrals 43–47, 45, including 9–21, 14 lanceolate humerals, 6–10, 8 non-lanceolate humerals, 14–19, 15 other non-lanceolate dorsocentrals, and 7–9, 8 lanceolate prescutellars. Preepisternum with 2–3, 3 setae in anterior row, and posterior with 3 setae. Acrostichals 2–6, 5; prealars 4–10, 7; supraalar 1; posterior anepisternum II with 2–4, 3 setae; epimeron II with 3 setae; scutellum with 6–9, 8 setae.

***Legs*.** Spur of fore tibia 44 µm, of mid tibia 17–19, 18 µm and 14–18, 16 µm long, of hind tibia 37–45, 41 µm and 13–22, 17 µm long. Tibial comb 42–45, 44 µm long, comb with 13 teeth. Width at apex of fore tibia 31–40, 35 µm, of mid tibia 31–33, 32 µm, of hind tibia 31–40, 35 µm. Lengths and proportions of legs in Table 2.

***Hypopygium*** (Fig. 8). “Anal point” broadly rounded, with 17–32 setae. Laterosternite IX with 3–5, 4 setae. Phallapodeme 43–62, 50 µm long; transverse sternapodeme 68–83, 77 µm long. Virga 32–35, 33 µm long, consisting of slender single spine. Gonocoxite 141–148, 144 µm long. Inferior volsella with developed digitiform dorsal lobe. Gonostylus 84–98, 91 µm long, with pointed crista dorsalis. Megaseta hair-like, 17–20, 19 µm long. HR 1.46–1.68, 1.59; HV 2.81–3.15, 2.95.

**Table 2.** Lengths (in µm) and proportions of leg segments of male *L. subtilus* Liu & Yan, sp. nov. (*N* = 6).

	fe	ti		ta_1_		ta_2_		ta_3_		ta_4_
p1	540–580, 560	690–730, 712		340–360, 352		200–230, 215		130–140, 132		80–90, 87
p2	590–620, 602	620–650, 632		270–290, 280		170–180, 178		120		60–80, 72
p3	590–630, 605	730–760, 745		420–440, 432		220		190–200, 192		95–100, 98
	ta_5_		LR		BV		SV		BR	
p1	80		0.49		3.04–3.25, 3.20		3.61–3.64, 3.62		1.84–2.00, 1.88	
p2	55–70, 64		0.44–0.45, 0.44		3.47–3.55, 3.49		4.38–4.48, 4.41		2.50	
p3	80		0.55–0.60, 0.58		2.97–3.05, 3.02		3.13–3.16, 3.14		2.00–2.60, 2.38	

#### Remarks.

The characters of the new species mainly agree with *L. eltoni* (Edwards, 1922) (Sæther 1990b). However, the new species differs from the latter on the basis of following characters: (1) the obvious costal extension of the new species (22–40 µm), shorter than for *L. elotoni* (77–94 µm); (2) “Anal point” of *L. eltoni* strongly projecting with apical notch, whereas in the new species it is not notched; (3) the new species has hair-like megaseta in the gonostylus, whereas the megaseta of *L. eltoni* is spine-like, subapically.

#### Distribution.

The species were collected in a subtropical mountain area in Sichuan Province in Oriental China.

### Key to adult males of *Limnophyes* from China

**Table:** 

1	Preepisternum with dorsocentral to posterocentral group of setae, no anterior setae	***L. brachytomus* (Kieffer)**
–	Preepisternum with anterior row of setae, with or without additional dorsocentral to posterocentral setae	**2**
2	Dorsocentral without lanceolate humerals and/or prescutellars	**3**
–	Dorsocentral with lanceolate humerals and/or prescutellars	**6**
3	“Anal point” strongly bifid, inferior volsella triangular	***L. verpus* Wang & Sæther**
–	“Anal point” not bifid, inferior volsella not triangular	**4**
4	First abdominal segment pale	***L. palleocestus* Wang & Sæther**
–	First abdominal segment brown	**5**
5	Inferior volsella very small or absent	***L. minerus* Liu & Yan, sp. nov.**
–	Inferior volsella moderately large, rectangular	***L. minimus* (Meigen)**
6	More than 13 lanceolate humerals and lanceolate prescutellars combined	**7**
–	Less than 10 lanceolate humerals and lanceolate prescutellars combined	**11**
7	Virga very slender, anal lobe slightly projecting	***L. subtilus* Liu & Yan, sp. nov.**
–	Virga not slender, anal lobe reduced or right angled	**8**
8	Lanceolate prescutellars absent	***L. pumilio* (Holmgren)**
–	Lanceolate prescutellars present	**9**
9	Megaseta long, bristle-like	***L. opimus* Wang & Sæther**
–	Megaseta absent or hair-like	**10**
10	“Anal point” apically notched, AR 0.49–0.79	***L. pentaplastus* (Kieffer)**
–	“Anal point” apically not notched, AR 0.18–0.30	***L. gurgicola* (Edwards)**
11	Gonostylus with strongly triangular protrusive middle part	***L. triangulus* Wang**
–	Gonostylus without such strongly triangular protrusive middle part	**12**
12	Gonostylus with rounded crista dorsalis	***L. orbicristatus* Wang & Sæther**
–	Gonostylus often with pointed crista dorsalis	**13**
13	“Anal point” pronounced parallel-sided, dorsal lobe of inferior volsella triangular	***L. habilis* (Walker)**
–	“Anal point” not parallel-sided, dorsal lobe of inferior volsella not triangular	**14**
14	Thorax with scalpellate acrostichals, virga with 3 spines	***L. aquamatus* Andersen**
–	Thorax without scalpellate acrostichals, virga with 1 or 2 spines	**15**
15	Flagellum with 11–12 flagellomeres, a strong tubercle in place of humeral pit	***L. bullus* Wang & Sæther**
–	Flagellum with 13 flagellomeres, humeral pit not as above	**16**
16	Virga consisting of two fused spines, “Anal point” relatively small, broad-based, bluntly triangular	***L. nudus* Song, Zheng, Wang & Qi**
–	Virga consisting of one simple spine, “Anal point” strong to moderately projecting with or without apical notch	***L. difficilis* Brundin**

## Supplementary Material

XML Treatment for Limnophyes minerus

XML Treatment for Limnophyes minimus

XML Treatment for Limnophyes subtilus
